# Determination of the Main Phase Transition Temperature of Phospholipids by Oscillatory Rheology

**DOI:** 10.3390/molecules28135125

**Published:** 2023-06-29

**Authors:** Lívia Budai, Marianna Budai, Tamás Bozó, Gergely Agócs, Miklós Kellermayer, István Antal

**Affiliations:** 1 Department of Pharmaceutics, Semmelweis University, Hőgyes Str. 7, 1092 Budapest, Hungary; budai.livia@semmelweis.hu (L.B.); budaimarianna@gmail.com (M.B.); 2 Department of Biophysics and Radiation Biology, Semmelweis University, Tűzoltó Str. 37–7, 1094 Budapest, Hungary; bozo.tamas@med.semmelweis-univ.hu (T.B.); agocs.gergely@med.semmelweis-univ.hu (G.A.); kellermayer.miklos@med.semmelweis-univ.hu (M.K.)

**Keywords:** phospholipid, phase transition temperature, oscillatory rheology, complex viscosity, reverse-type phospholipid complex

## Abstract

Knowledge of the physical and chemical properties of phospholipids, such as phase transition temperatures (Tc), is of great importance in order to reveal the functionalities of biological and artificial membranes. Our research group developed an oscillatory rheological method for the simple and rapid determination of phase transition temperatures (Tc). The phospholipids constructing the membranes undergo conformational changes at their Tc, which cause alterations of viscoelastic properties of the molecules. The oscillatory technique recommended by us proved to be appropriate to reveal the altered molecular properties of phospholipids as tracking the slightest changes in the viscoelasticity. Our study demonstrates the abrupt changes in rheological properties at Tc for the following phospholipids: 1,2-Dimyristoyl-sn-glycero-3-Phosphocholine (DMPC), 1,2-Dipalmitoyl-sn-glycero-3-Phosphatidylcholine (DPPC), and 1,2-Distearoyl-sn-glycero-3-Phosphocholine (DSPC), proving that the applied methodology is adequate for determining the Tc of phospholipids.

## 1. Introduction

Determination of the phase transition temperatures (Tc) of phospholipid molecules is of crucial interest in many research fields of biological, pharmaceutical, and technological development. In numerous applications, from permeability studies to the development of drug delivery systems, phospholipids, as biocompatible molecules, can be widely used [1]. In the development of liposomal drug carriers, phospholipids are the main building molecules contributing to the successful formation of nanosized delivery systems. Therefore, the physical properties of phospholipids are of great importance. The formulation of thermosensitive liposomes represents a special field of pharmaceutical development which has gained great attention in the targeting of drug molecules [2].

Composed of phospholipids, lipid nanoparticles (LNPs) are considered the best candidates for nucleic acid delivery (e.g., mRNA delivery in vaccines). Cationic lipids, ionizable lipids, polyethylene glycols (PEGs), and cholesterol are involved in the construction of LNPs, indicating the significant role of phospholipids in the formulation [3,4]. The physical properties of phospholipids—including the main phase transition temperature—are closely related to permeability and drug release; therefore, the determination of their temperature-dependent parameters is essential for the development of biomimetic membranes and phospholipid-based drug delivery systems such as liposomes or LNPs.

Determination of the main phase transition temperature of lipids can be carried out with selected techniques such as differential scanning calorimetry (DSC) [5,6], fluorescence depolarization [7,8], nuclear magnetic resonance spectroscopy (NMR) [9,10], Fourier-transform infrared spectroscopy (FTIR) [11,12], atomic force microscopy (AFM) [13,14,15,16], dynamic light scattering (DLS) [17], calcein release measurements [2], or nanoplasmonic sensing (NPS) [18]. However, these techniques can be fraught with difficulties. In some cases, the sample preparation could be a time-consuming step, e.g., DSC techniques require the formulation of liposomes from phospholipids prior to Tc determination. Liposome formulation including lipid film preparation and hydration is a time-consuming process and could be difficult to reproduce [5,6]. It is also clear that in case of some of the above methods, quite complex instrumentation is needed [9,10], or the modification of phospholipid molecules (e.g., the addition of fluorescent membrane probes [7,8] or calcein) is required for the measurements [2]. On this basis, novel techniques offering simple ways for the determination of phase transition temperatures are of great interest. Our research focused on the determination of phase transition temperatures using oscillatory rheology.

## 2. Results and Discussion

### 2.1. Rheological Measurements

The phase transition temperatures for the pre and main transitions of the examined lipids (DMPC, DPPC, and DSPC) were determined from the literature. These data were determined earlier using various methods; selected results are collected in Table 1, Table 2 and Table 3. Thus, in the cases of all examined lipids, the expected phase transition temperatures were known. During our experiments, these expected temperatures were used as reference values for comparison with the values gained by our methodology.

#### 2.1.1. Rheological Experiments of Liposomal Dispersions

According to the measurements on liposomal dispersions, the oscillatory rheology method does not seem to be appropriate for determining the phase transition temperatures of lipids. Our temperature-dependent rheological measurements (complex viscosity as a function of temperature) on liposomal dispersions showed no changes at the temperatures of the expected main phase transitions (Figure S1 in Supplementary Materials). This observation can be explained by the unique structure of the liposomes. The structure of liposomes hides the fatty acids of phospholipids inside the phospholipid bilayer, obscuring the accurate detection of the slight changes in their mobility and viscoelasticity during rheological experiments. In the phospholipid bilayer of a liposome, the fatty acids are located in the inner part of the bilayer that has no direct contact with the environmental medium, masking the rheological changes in fatty acid tails. Liposomes have a high elasticity and deformability; therefore, the detection of viscoelastic changes in the fatty acids placed in the inner part of the bilayer is unlikely.

#### 2.1.2. Rheological Experiments of Reverse-Typed Micelles

In order to overcome the “shielding effect” of liposomal bilayers, and ensure the accurate detection of flow properties, the fatty acids should be turned to the outside, making direct contact with the solvent molecules. Thus, the preparation of reverse-typed micelles using apolar solvents such as vegetable oils (phospholipids dispersed in oil) was attempted.

Surprisingly, the rheological analysis of these reverse-type micelles showed no changes at the expected temperatures (Figure S2 in Supplementary Materials). It can be assumed that determining the rheological properties of phospholipids requires a kind of assemblance of the molecules providing coordinated movement of the fatty acids. The absence of detectable phase transition temperatures in the case of reverse-type micelles may be explained by the relatively small size of micelles, making the ordered, coordinated, extensive co-movement of fatty acid tails impossible.

#### 2.1.3. Rheological Experiments of Reverse-Type Phospholipid Complexes (RTPCs)

In order to overcome the problem posed by the small size of micelles, reverse-type phospholipid complexes (RTPCs) offering larger surfaces for the co-movement of lipids were prepared. It can be concluded that for the success of the rheological measurements, a remarkable accumulation of phospholipids is required, with the fatty acids orienting forward to the external environmental medium rather than to the interior of the bilayers. The criteria can be fulfilled by the preparation of RTPCs, which can be produced through the addition of water to the oily phospholipid mixture. The microscopic and rheological analyses of such RTPCs are presented subsequently.

The temperature-dependent rotational measurements of RTPCs showed no appreciable change in viscosity at the expected phase transition temperatures of phospholipids. It can be assumed that in the case of rotational measurements, the rotational shear force causes excessive strain changes in the structure of RTPCs, thereby over-riding the detectable changes associated with the phase transition.

However, in contrast to rotational experiments, oscillatory measurements demonstrated apparent and abrupt changes for RTPC samples at the expected temperatures. The elastic modulus (G′), viscous modulus (G″), complex modulus (G*), and complex viscosity (η*) were detected as a function of temperature.

#### 2.1.4. Evaluation of Oscillatory Measurements of Reverse-Type Phospholipid Complexes (RTPCs)

The sinusoidal oscillation of the materials examined in oscillatory rheology can be described by the viscosity and elasticity moduli. The viscous modulus is related to the internal friction between the molecules during the deformation and characterizes the liquid-like behavior component. The elastic modulus represents the solid-state behavior of a material which makes it more resistant against deformation. The complex modulus can be described as the overall resistance to deformation, which includes both the viscous and elastic moduli. The viscous and elastic moduli enable the calculation of complex viscosity, where ω means the angular frequency of the oscillation [19].

(1) $$\left|\eta^{*}\right|=\sqrt{{\left(\frac{G\prime \prime }{\omega }\right)}^{2}+{\left(\frac{G\prime }{\omega }\right)}^{2}}=\frac{\left|G^{*}\right|}{\omega }$$

All of the mentioned oscillatory parameters clearly indicate the slight alterations in elasticity as a consequence of the changed mobility of fatty acids.

The complex viscosity (η*) includes both the viscous and elastic moduli of oscillation; thus, it can characterize the alterations in a temperature-dependent oscillatory measurement the best. Therefore, for the further analysis of measurements and the evaluation of intersection points of the trendlines, the complex viscosity was chosen from the oscillatory parameters.

Sudden changes in the oscillatory parameters can be observed at the expected phase transition temperatures in the RTPC samples. The abrupt changes in the parameters divide the oscillatory curves into three phases, which are referred to as the lower, middle, and upper phases. The lower and the uppers phases can be assigned to the parameters measured at lower and higher temperatures, respectively. The middle phase represents the sudden change in the oscillatory parameters in the region among the two abovementioned phases. Three straight lines exhibiting different slopes can be assigned to the three phases of all charts. The resulting intersection points of the three trendlines (two intersection points per chart) indicate the range of phase transitions. The middle phase, representing the transition of the phospholipids from fluid into rigid state, manifests in a significant change in the elastic and viscous moduli and, as a consequence, the alteration of the complex modulus and complex viscosity. The fluid state of the phospholipids can be described with low oscillatory parameters, and the rigid state at lower temperatures results in high oscillatory parameters, indicating the increased elasticity of the reversed phospholipid complexes.

The first method used for the determination of phase transition temperature was differential scanning calorimetry (DSC) based on the tracking of thermodynamical changes as a function of the temperature [5]. The absorbed heat is recorded as heat flow during DSC, and the main phase temperature can be attributed to the maximum heat flow detected. A possible drawback of the DSC method is that the determination of phase transitions is based purely on thermal properties, disregarding any conformational changes or alterations in the elasticity of the structure.

Based on temperature-controlled atomic force microscopy (AFM), the height differences of phospholipid bilayers can be analyzed. The thickness of the phospholipid bilayer can be determined by measuring the height contrast of differently colored areas [13,14,15,16]. Among its disadvantages are that the AFM technique requires the time-consuming imaging analysis of liposomal preparations at different temperatures, which does not provide the presence of Tc as a sharp-edged and unambiguous transition.

From the calcein release measurements, it can be assumed that permeability changes in DPPC liposomes occur at temperatures lower than the Tc measured by DSC [2]. The difference raises the possibility that the permeability and mobility changes in phospholipids are not fully consistent with the alterations of thermal properties. The application of other techniques makes it possible to describe the physical changes in phase transition more accurately.

The nanoplasmonic sensing technique overcomes the mentioned deficiency and investigates the conformational changes in liposomes during the phase transitions [18].

With the use of the oscillatory rheology recommended by our group, the transition from a fluid into a rigid state can be examined based on the changes in viscoelasticity. The ordered assemblance of the phospholipids in reverse-type complexes produces structures in which the mobility changes in phospholipids can accurately be followed. By applying constant oscillation frequency and constant amplitude, the rheological parameters will only depend on the temperature. Changes in the flow resistance can be detected by measuring the elastic and viscous moduli as well as the complex modulus and complex viscosity.

**Figure 1:**
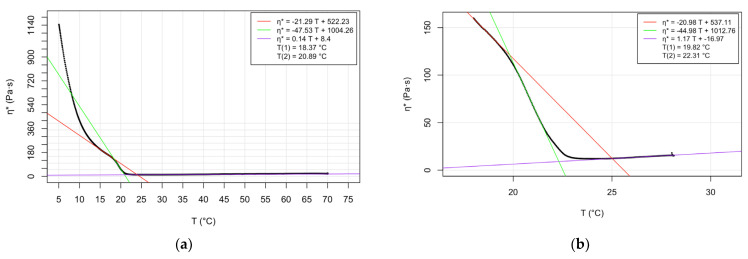

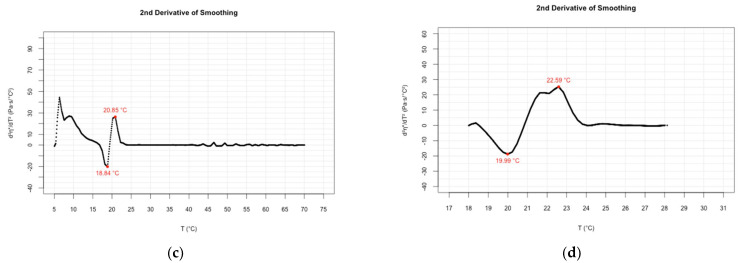
Temperature dependence of complex viscosity of DMPC: (**a**) raw data at 3 °C/min cooling rate; (**b**) raw data at 1 °C/min cooling rate; (**c**) second derivative of data at 3 °C/min cooling rate; and (**d**) second derivative of data at 1 °C/min cooling rate.

**Figure 2:**
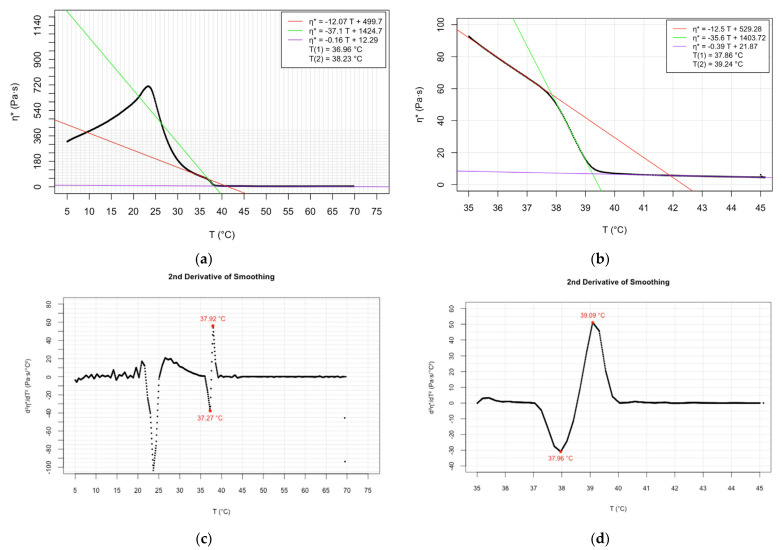
Temperature dependence of complex viscosity of DPPC: (**a**) raw data at 3 °C/min cooling rate; (**b**) raw data at 1 °C/min cooling rate; (**c**) second derivative of data at 3 °C/min cooling rate; and (**d**) second derivative of data at 1 °C/min cooling rate.

**Figure 3:**
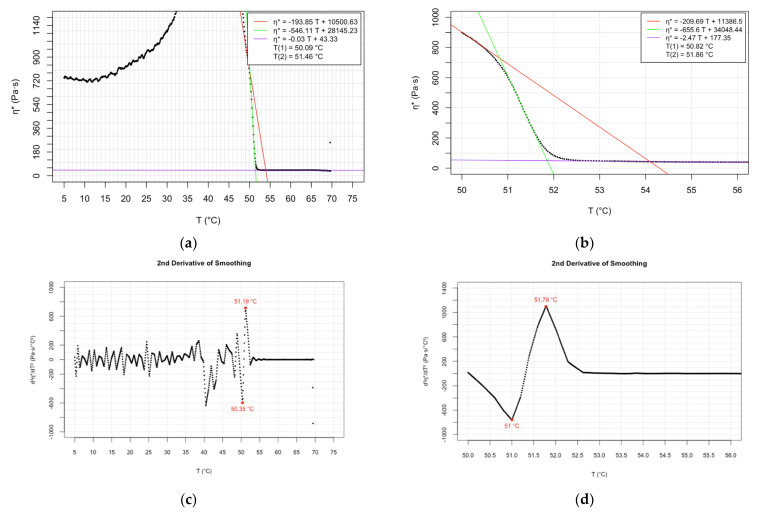
Temperature dependence of complex viscosity of DSPC: (**a**) raw data at 3 °C/min cooling rate; (**b**) raw data at 1 °C/min cooling rate; (**c**) second derivative of data at 3 °C/min cooling rate; and (**d**) second derivative of data at 1 °C/min cooling rate.

The newly determined main phase transition temperatures are presented in detail in the graphs (Figure 1, Figure 2 and Figure 3) and Supplementary Materials (Figures S3–S8). The sub-figures (a) and (b) of the graphs (Figure 1, Figure 2 and Figure 3) depict the complex viscosity obtained as raw data as a function of temperature applying at a cooling rate of 3 °C/min over a range of 5–70 °C and experiments at lower cooling speed (1 °C/min), respectively. The parallel measurements of the samples demonstrating the raw data can be found in Supplementary Materials (Figures S3–S8). For the sake of comparison, first, derivatives of the temperature-dependent complex viscosity of DMPC at 3 °C/min cooling rate are shown in graph in the Supplementary Materials (Figure S9). More spectacular are the experiments of second derivate data which interpret the phase transition temperatures as readable peak values. Plots of second derivative data corresponding to cooling rates of 3 °C/min and 1 °C/min are shown in the graphs as sub-figures (c) and (d), respectively (Figure 1, Figure 2 and Figure 3). Plots of parallel measurements of the second derivative of the temperature-dependent complex viscosity for DMPC, DPPC, and DSPC are presented in the Supplementary Materials (Figures S10–S12).

As shown in Table 1, Table 2 and Table 3 the DSC [5,6] and fluorescence depolarization methods [7,8] detect two phase transition temperatures, namely, the pretransition and the main transition. In addition to these two temperatures the nanoplasmonic sensing provides a third transition temperature based on conformational changes in the phospholipids [18] (Table 1, Table 2 and Table 3).

The evaluation of the oscillatory rheological experiments provides two transition temperature values, indicating the beginning and the completion of the phase transition process. Regression lines can be ordered to the three phases of a temperature-dependent measurement. The intersection point of the lower and middle phase trendlines indicates the beginning of the transition process. The completion of the transition process is calculated as the intersection point of the middle and upper phase regression lines.

Comparing the phase transition data known from the literature with the data gained by our methodology, we have to take into consideration that the samples for the oscillatory rheology were prepared in oil and contained just trace amounts of water to enable the formation of reverse-type complexes. The slightly lower Tc values compared with previously determined DSC data can be explained by the application of oil as a solvent in our samples. The sunflower oil applied as a solvent has higher viscosity than aqueous solvents, which can contribute to the slower adaptation of changes in molecular viscoelasticity during cooling processes.

By comparing the Tc values obtained at the two different cooling rates applied, we can conclude that the higher Tc values obtained correlated to the slower cooling process (Table 1, Table 2 and Table 3). The relationship mentioned was the least pronounced in the case of DSPC (Table 3). It is assumed that a slower cooling process provides the phospholipids with a more long-lasting opportunity for adaption to the changed environmental conditions, including their viscoelasticity. The longer adaptation time results in the slightly higher Tc values.

In contrast to the drawbacks of the methods previously used for the determination of phase transition temperatures, the oscillatory rheological technique—tested and recommended by us—needs no modification of the lipid molecules, and the experiments can easily be performed. Furthermore, oscillatory measurements carried out in the linear viscoelastic region do not destroy structural changes in the samples. Moreover, evaluations of the temperature-dependent oscillatory data are simple and clear [19].

### 2.2. Microscopic Analysis

**Figure 4:**
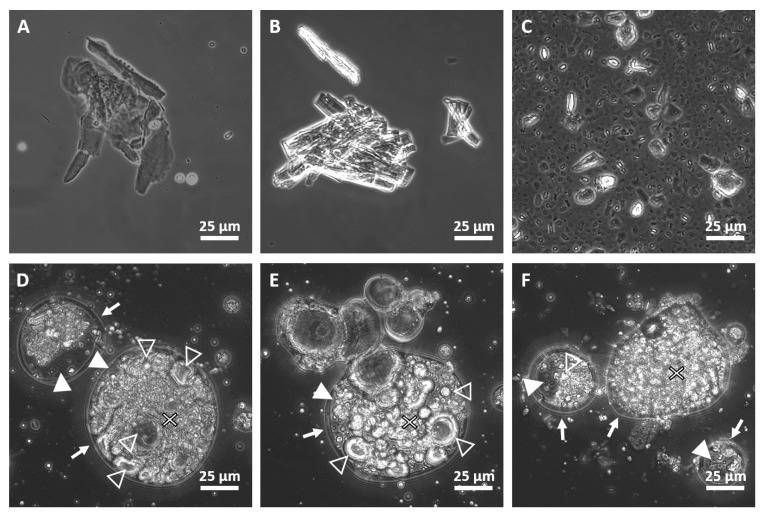
Phase-contrast microscographs of phospholipid–oil mixtures (upper panels) and reverse-type phospholipid complexes (bottom panels). DMPC, DPPC, and DSPC are dispersed in oil in panels (**A**–**C**), respectively. (**D**–**F**) show RTPCs made of DMPC, DPPC, and DSPC, respectively. Arrows mark the wall structure; solid triangles indicate inner segments; empty triangles denote rounded objects; x-s show layered, densely packed areas.

Phospholipids dispersed in sunflower oil appeared as amorphous solid particles in the phase-contrast microscopy images (Figure 4A–C). The addition of a small amount of water followed by vigorous stirring instantaneously precipitated the samples, i.e., their opacity increased and white fluffy floccules appeared in them. Phase-contrast micrographs of these samples showed rounded structures of diverse size (Figure 4D,E), which we coined as reverse-type phospholipid complexes (RTPCs, see explanation below). These complexes have a peculiar structure that may be characterized by four main features: (1) they are surrounded by a wall; (2) they contain low-contrast areas exhibiting the same grayness and homogeneity as the surrounding area of the complexes, (3) they contain high-contrast areas that consist of rounded objects either being compact or encircling a low-contrast area, and (4) they contain densely packed layered and curved parts that resemble to the gyri and sulci of the brain (Figure 4D,E). These characteristics are hypothesized to be the result of hydration of the phospholipid head groups. Namely, walls may be single or multiple phospholipid layers encircling other lipid structures and oil inclusions (high- and low-contrast areas, respectively). Considering that the outer phase is oil, the layers must have an orientation inverse to conventional bilayers, i.e., the apolar chains point outwards and the head groups are found in the inner plane of the bilayer. Lipid structures may consist of vesicles that are packed and multilamellar, as their thick wall suggests. The low water content suggests that there are no large bulk water reservoirs in the system, and the non-structural water may be distributed in small, reversed micelles and/or confined between adjacent planar phospholipid head group layers. Therefore, the large, circular, low-contrast core regions of rounded objects are possibly oil phases inside a reversed multilamellar vesicle. It is the sulci- and gyri-like regions that fill the vast majority of the complexes. These parts may be stacked, inversely oriented multilayer lipid assemblies that fold and meander to form a packed structure.

**Figure 5:**
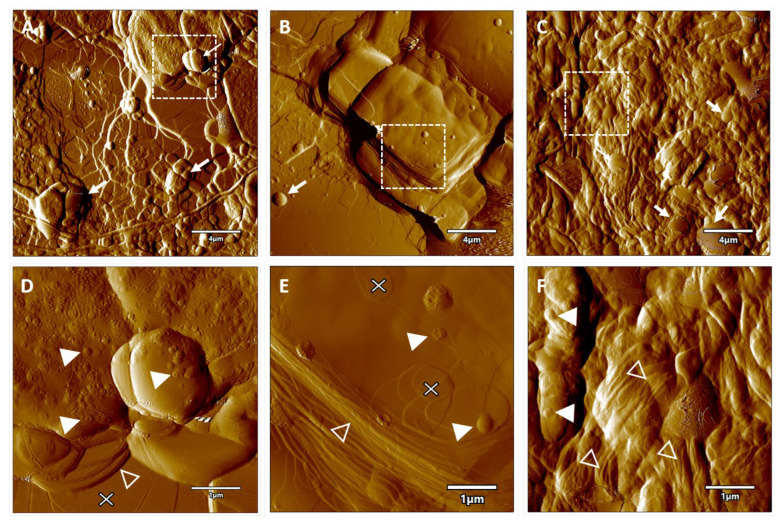
The 20 × 20 μm AFM amplitude contrast images of reverse-type (**A**) DMPC, (**B**) DPPC, and (**C**) DSPC phospholipid complexes. (**D**–**F**) show close-ups of the areas marked with white dashed rectangles in (**A**–**C**), respectively. Arrows mark large, rounded structures; solid triangles indicate circular objects; empty triangles denote stack edges; x-s show patches.

**Figure 6:**
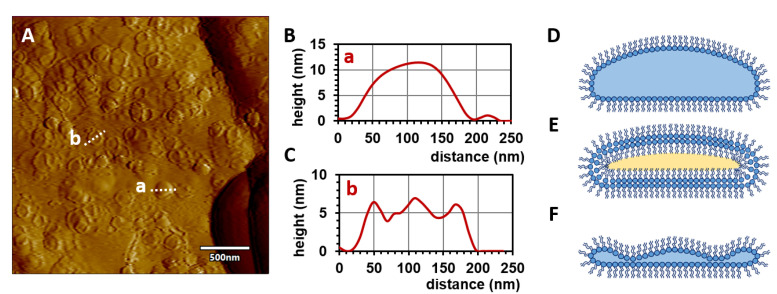
(**A**) AFM amplitude contrast image of the surface of a reverse-type DMPC phospholipid complex (an area from Figure 5D). (**B**,**C**) Height section profiles taken alongside dotted lines a and b in panel (**A**). (**D**,**E**) Schematic figures (not to scale) of an inverse micelle and inverse vesicle corresponding to height profiles in (**B**). (**F**) A scheme of a flattened inverse micelle according to the height profile in (**C**). A light blue interior represents water; yellow represents oil.

To observe the structures at higher resolution, AFM experiments were carried out. Figure 5 shows the surface of RTPCs from different lipids. Four distinct structural features can be identified. (1) Planar patch-like regions and (2) piled edges are the signs of individual and stacked planar phospholipid layers, respectively. (3) Larger rounded objects may be vesicles surrounded by single or multiple phospholipid layers. As there is not enough water in the system to fill the large vesicles, these objects may contain oil, which means that the lipid layers should exhibit inverse orientations (i.e., apolar chains point toward the core and head groups orient toward each other to form a bilayer). (4) Small circular objects (d < 200 nm) were also found, showing either rounded or rimmed morphology (Figure 6A). The maximum height of the round objects was approximately 12 nm (Figure 6B); therefore, these objects may either be inverse micelles with an aqueous core or inverse, oil-filled vesicles (Figure 6D,E). As they had a relatively flat shape, these micelles/vesicles could originally have been disc-like, or they were partially flattened on the surface due to adhesion forces. Notably, the rimmed objects had a central protruding part. The maximum height of the rim and the central part was approximately 7 nm, while the minimum height of the vesicle was about 4 nm (Figure 6C). This latter dimension corresponds to the thickness of a lipid bilayer; thus, it is plausible that the characteristic shape of these objects is a result of the advanced flattening of an inverse micelle (Figure 6F). Therefore, it suggests that the small circular objects reflect different phases of micelle flattening.

Overall, AFM images confirmed that vesicles, multilayer lipid assemblies, and micelles appear in phospholipid–oil systems upon the addition of a small amount of water—as was hypothesized based on phase-contrast micrographs. Furthermore, analysis of the cross-sectional height profile of micelles provided indirect evidence for the reverse orientation of phospholipids in their wall.

## 3. Materials and Methods

### 3.1. Materials

1,2-Dimyristoyl-sn-glycero-3-Phosphocholine (DMPC), 1,2-Dipalmitoyl-sn-glycero-3-Phosphatidylcholine (DPPC), and 1,2-Distearoyl-sn-glycero-3-Phosphocholine (DSPC) as crystalline solid powders were purchased from the Cayman Chemical Company (Budapest, Hungary). The purity of lipids was at least 98%. The sunflower oil (*Helianthi annui oleum raffinatum*, Ph.Eur.) was obtained from Hungaropharma Ltd. (Budapest, Hungary). Deionized ultrafiltered water was produced using the Milli-Q system (Millipore Inc., Budapest, Hungary).

### 3.2. Preparation of Samples

#### 3.2.1. Preparation of Liposomal Dispersions

Multilamellar vesicles (MLVs) were prepared by applying the thin-film hydration technique. From a stock solution of lipid (20 mg/mL lipid in absolute ethanol), the appropriate volume was taken, and the solvent was evaporated under nitrogen stream and held thereafter in a vacuum desiccator for 24 h. The hydration of the lipid films was performed at 60 °C, above the main phase transition temperature of liposomal samples used in the present study. Liposomal samples were hydrated with deionized, ultrafiltered water. The final lipid concentration was 15.0 mg/mL.

#### 3.2.2. Preparation of Reverse-Type Micelles

Subsequently, 100 mg of phospholipid (DMPC, DPPC, or DSPC) was measured in a tube, and 400 μL of sunflower oil was added. The sample was vigorously mixed using a vortex mixer for 1 min. The samples were heated in a water bath above their mean phase transition temperature.

#### 3.2.3. Preparation of Reverse-Type Phospholipid Complexes (RTPCs)

The RTPCs were prepared based on the lecithin organogel preparation procedure [20]. For this, 100 mg of phospholipid was measured in a tube and 400 μL of sunflower oil was added. The sample was vigorously mixed using a vortex mixer for 1 min. The oils containing phospholipids were heated in a water bath above their mean phase transition temperature. At elevated temperature, 15 μL deionized, ultrafiltered water was added, and the mixture was vortex-mixed again for 1 min. The addition of water resulted in cloudy flocculation in the oily sample. The prepared sample was immediately measured using a rheometer.

### 3.3. Rheological Experiments

The oscillatory measurements were carried out with a Kinexus Pro rheometer (Malvern Instruments Ltd., Malvern, UK). Data were registered in rSpace for Kinexus Pro 1.5 software (Netzsch, Bayern, Germany). Cone and plate geometry was used with code number CP 4/40 SR0207SS PL65 SO815SS. The gap between the cone and plate of sample placement was 0.1500 mm. The temperature was controlled using the Peltier system of the rheometer with an accuracy of ±0.1 °C. Rotational measurements were carried out in a temperature range of 5–70 °C using the same geometries and applying 1 Pa as shear stress. The temperature-dependent oscillatory sweep tests were recorded in two methodologies: either from 70 °C to 5 °C, during which the samples were cooled down at a cooling speed of 3 °C/min, or in a cooling process at a cooling speed of 1 °C/min in an approximately 20 °C narrow temperature range, which implied the requested Tc values. A strain amplitude of 1% and an oscillation frequency of 1 Hz were set in all measurements. In all experiments, a cylindrical cover made of stainless steel was placed over the tests in order to create a closed environment around the sample. In each sample, two parallels were measured and analyzed.

### 3.4. Data Analysis for Rheological Measurements

Analysis was carried out in R (R Core Team 2022, v4.1.3, Vienna, Austria). The complex viscosity was plotted as a function of the temperature [21]. Linear segments of the individual phase regions were manually selected and each fitted with a linear model. Slopes and y-axis intercepts, as well as their standard errors (SEs), were extracted from the fitted models. Phase transition temperatures were determined by finding the intersections of the fitted linear slopes.

### 3.5. Phase-Contrast Microscopy

Freshly prepared RTPCs were left to cool down to room temperature, and 10 μL was pipetted on a clean microscope slide and covered with a coverslip. These samples were imaged with a Nikon Eclipse Ti-U inverted microscope (Auro-Science Ltd., Budapest, Hungary) using a 20× Nikon S Planfluor phase-contrast objective. Micrographs were recorded using a Jenoptik Progres Gryphax Rigel microscope camera (Grimas Ltd., Budapest, Hungary).

### 3.6. Atomic Force Microscopy (AFM)

For AFM imaging, 10 μL RTPC was dropped on freshly cleaved mica. Another mica sheet was placed on it and squeezed gently by hand until the sample formed a thin film between the sheets. After 10 min, the sheets were separated and the residual oil droplets were blotted from the side using a paper wipe (Kimtech Science * Precision Wipe, Kimberley-Clark Co., Irving, TX, USA). Samples were imaged with an MFP 3D atomic force microscope (Asylum Reserach, Santa-Barbara, CA, USA) with a 0.2–0.4 Hz line-scanning rate in air. A silicon cantilever (OMCL AC-160TS, Olympus, Tokyo, Japan) was used in non-contact mode, oscillated at its resonance frequency (300–320 kHz, typically). Images with 512 × 512 pixel dimensions were collected and analyzed using built-in algorithms of the AFM driver software (IgorPro, WaveMetrics, Inc., Lake Oswego, OR, USA).

## 4. Conclusions

To the best of our knowledge, the present study demonstrates, for the first time, that oscillatory rheology is a valuable technique for the determination of the phase transition temperatures of phospholipids.

Sample preparation prior to oscillation measurements is simple. Instead of the time-consuming formulation of liposomes, reverse-type phospholipid complexes are prepared. Their viscoelastic properties can be precisely detected since the fatty acids of co-moving phospholipids protrude outwards.

The remarkable changes in viscoelastic properties of phospholipids can be unambiguously attributed to their phase transitions. The microscopic appearance of RTPCs reflects the heterogeneity of the samples, confirming the presence of multilayer lipid assemblies.

The phase transition temperatures determined by oscillatory rheology are in good arrangement with data measured previously using other techniques and by other research groups.

## Figures and Tables

**Table 1:** Phase transition temperatures of DMPC determined by various methods—literature data and determined values by oscillatory rheology based on raw data.

Method	Pretransition T_c1_ (°C)	Main Transition T_c2_ (°C)	Upper Transition T_c3_ (°C)	Reference
Differential scanning calorimetry	14.2	23.9		[5]
Cis-parinaric fluorescence intensity	10	23		[7]
Differential scanning calorimetry	14.1	23.9		[8]
Fluorescence depolarization	10; 12.4	23.8		[8]
Atomic force microscopy		26–27.5		[16]
Nanoplasmonic sensing	16.8	20.7	22.5	[18]
Oscillatory rheology (cooling speed 1 °C/min)	19.82; 19.87	22.31; 22.33		Present study
Oscillatory rheology (cooling speed 3 °C/min)	18.37; 18.59	20.89; 21.14		Present study

**Table 2:** Phase transition temperatures of DPPC determined by various methods—literature data and determined values by oscillatory rheology based on raw data.

Method	Pretransition T_c1_ (°C)	Main Transition T_c2_ (°C)	Upper Transition T_c3_ (°C)	Reference
Differential scanning calorimetry	35.3	41.4		[5]
Cis-parinaric fluorescence intensity	32	42		[7]
Differential scanning calorimetry	35.2	41.3		[8]
Fluorescence depolarization	22.9; 29.8; 31.9	40.6		[8]
Atomic force microscopy		42–52	53–60	[13]
Differential scanning calorimetry		42.37		[2]
Calcein release		40		[2]
Nanoplasmonic sensing	34.9	39.1	41.0	[18]
Oscillatory rheology (cooling speed 1 °C/min)	37.86; 37.91	39.24; 39.3		Present study
Oscillatory rheology (cooling speed 3 °C/min)	36.96; 37.0	38.23; 38.24		Present study

**Table 3:** Phase transition temperatures of DSPC determined by various methods—literature data and determined values by oscillatory rheology based on raw data.

Method	Pretransition T_c1_ (°C)	Main Transition T_c2_ (°C)	Upper Transition T_c3_ (°C)	Reference
Differential scanning calorimetry	51.5	54.9		[5]
Cis-parinaric fluorescence intensity	49	54		[7]
Differential scanning calorimetry	48.5	54.5		[8]
Fluorescence depolarization	43.2; 45.6	53.7		[8]
Nanoplasmonic sensing	46.7	51.7	55.5	[18]
Oscillatory rheology (cooling speed 1 °C/min)	50.82; 50.65	51.86; 51.57		Present study
Oscillatory rheology (cooling speed 3 °C/min)	50.09; 50.21	51.46; 51.43		Present study

## Data Availability

Not applicable.

## Supplementary Materials

The following supporting information can be downloaded at: https://www.mdpi.com/article/10.3390/molecules28135125/s1. Figure S1. Oscillatory measurement of DSPC liposomes; Figure S2. Oscillatory measurement of DMPC reverse-type micelles; Figure S3. Temperature dependence of the complex viscosity of DMPC (cooling speed 3 °C/min)—parallel measurement; Figure S4. Temperature dependence of the complex viscosity of DMPC (cooling speed 1 °C/min)—parallel measurement; Figure S5. Temperature dependence of the complex viscosity of DPPC (cooling speed 3 °C/min)—parallel measurement; Figure S6. Temperature dependence of the complex viscosity of DPPC (cooling speed 1 °C/min)—parallel measurement; Figure S7. Temperature dependence of the complex viscosity of DSPC (cooling speed 3 °C/min)—parallel measurement; Figure S8. Temperature dependence of the complex viscosity of DSPC (cooling speed 1 °C/min)—parallel measurement; Figure S9. First derivative of the temperature-dependent complex viscosity of DMPC at 3 °C/min cooling rate; Figure S10. Second derivative of the temperature-dependent complex viscosity of the DMPC parallel measurement (a) at a 3 °C/min cooling rate and (b) at a 1 °C/min cooling rate; Figure S11. Second derivative of the temperature-dependent complex viscosity of the DPPC parallel measurement (a) at a 3 °C/min cooling rate and (b) at a 1 °C/min cooling rate; Figure S12. Second derivative of the temperature-dependent complex viscosity of the DSPC parallel measurement (a) at a 3 °C/min cooling rate and (b) at a 1 °C/min cooling rate.

## Sample Availability

Samples of the compounds are available or not available from the authors.
