# Supplementary material for: Determination of the Main Phase Transition Temperature of Phospholipids by Oscillatory Rheology
Source: Molecules. 2023 Jun 29;28(13):5125. doi: 10.3390/molecules28135125 (PMC10343600; doi:10.3390/molecules28135125)
Supplement: Supplementary file 1 [file molecules-28-05125-s001.zip › molecules-2476441-supplementary.pdf]

Novel method for the determination of the main phase transition temperature by oscillatory rheology

Lívia Budai[1], Marianna Budai[1], Tamás Bozó[2], Gergely Agócs[2], Miklós Kellermayer[2], István Antal[1]

[1]Department of Pharmaceutics, Semmelweis University, Budapest, Hungary.

[2]Department of Biophysics and Radiation Biology, Semmelweis University, Budapest, Hungary.

Supplementary materials

Corresponding author:

István Antal PhD

Semmelweis University

Department of Pharmaceutics

Hőgyes E. u. 7., Budapest, H-1092, Hungary

Tel.: +36-1-476-3600/53016

Fax: +36-1-217-0914

E-mail: antal.istvan@semmelweis.hu

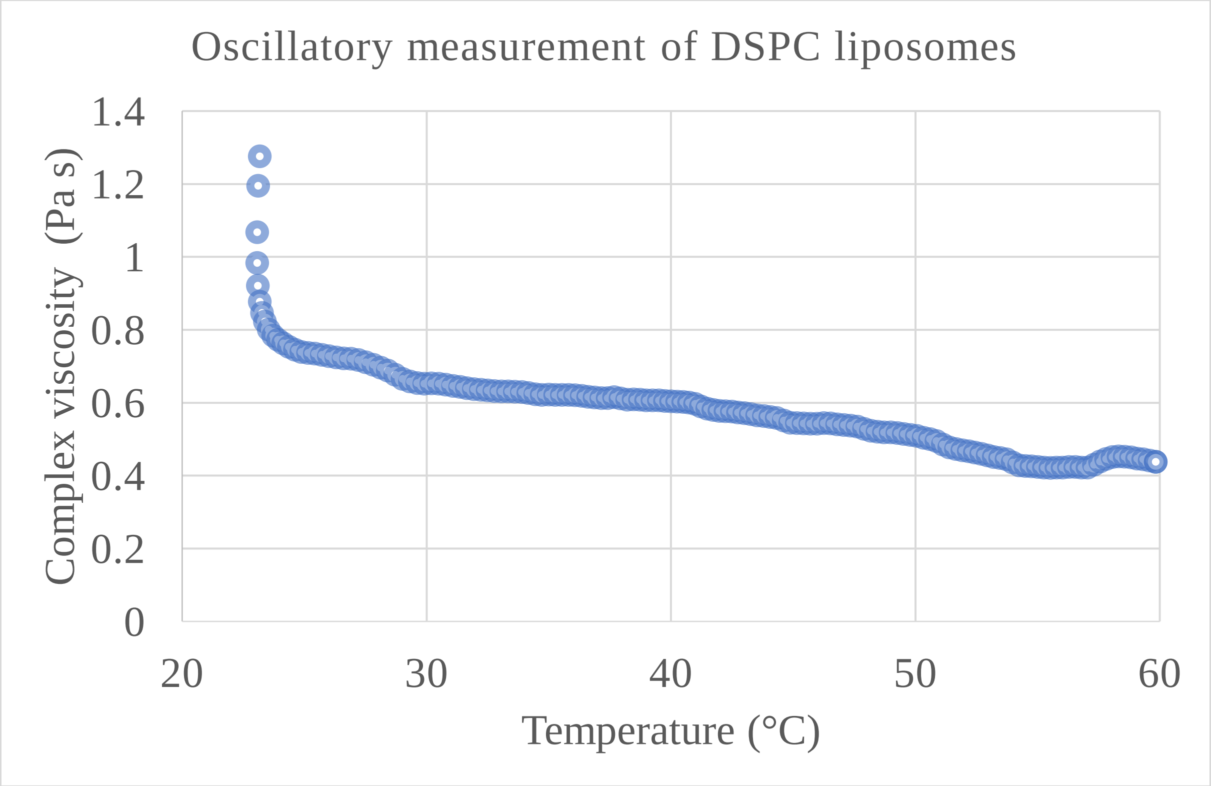

Figure S1. Oscillatory measurement of DSPC liposomes

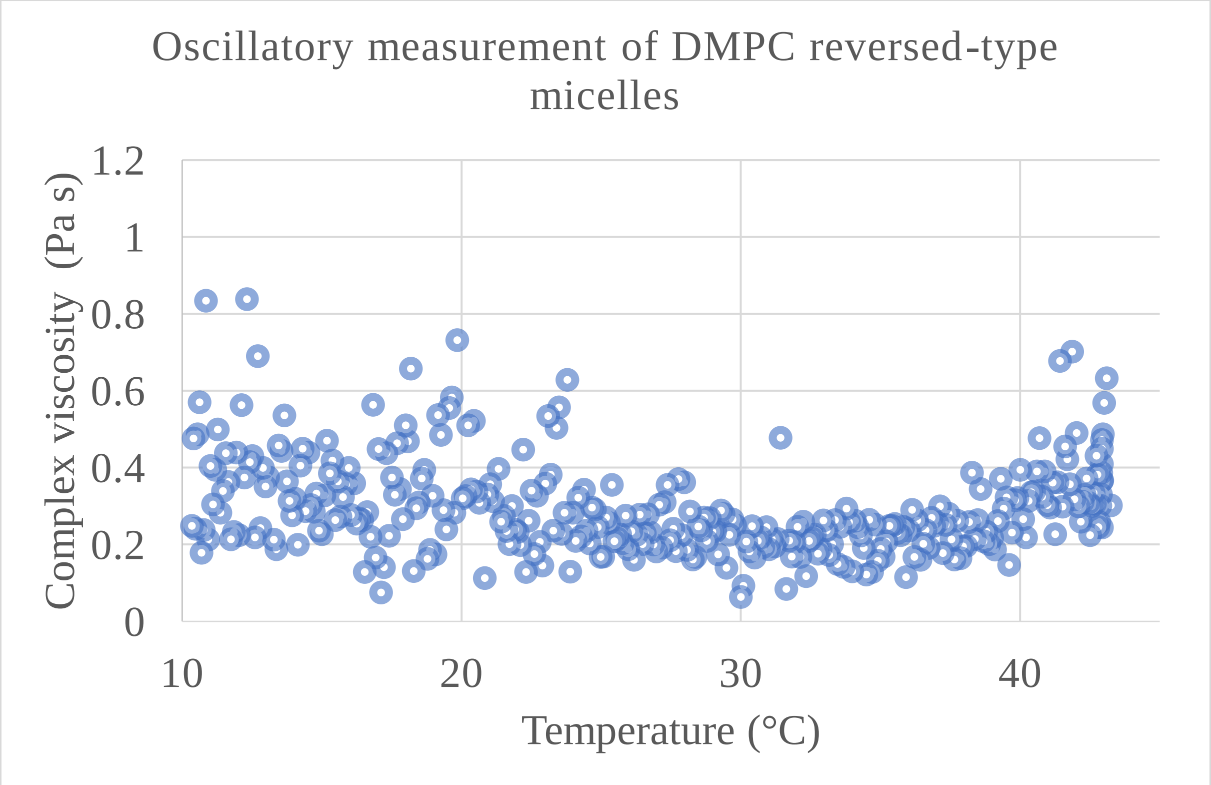

Figure S2. Oscillatory measurement of DMPC reversed-type micelles

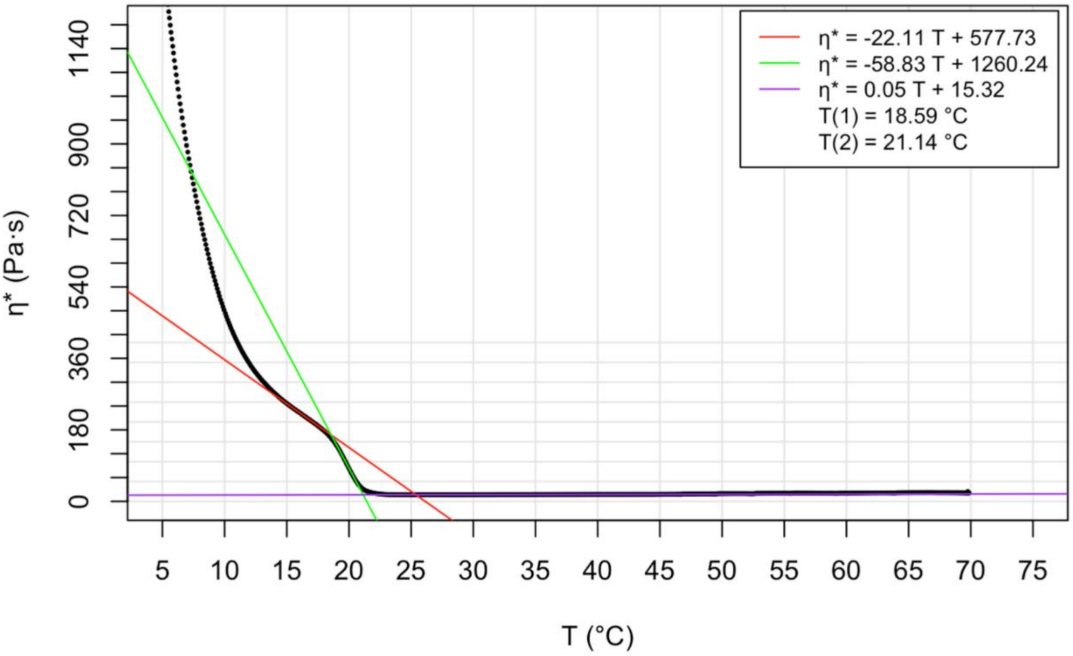

Figure S3. Temperature dependence of complex viscosity of DMPC (cooling speed 3 °C/min) - parallel measurement

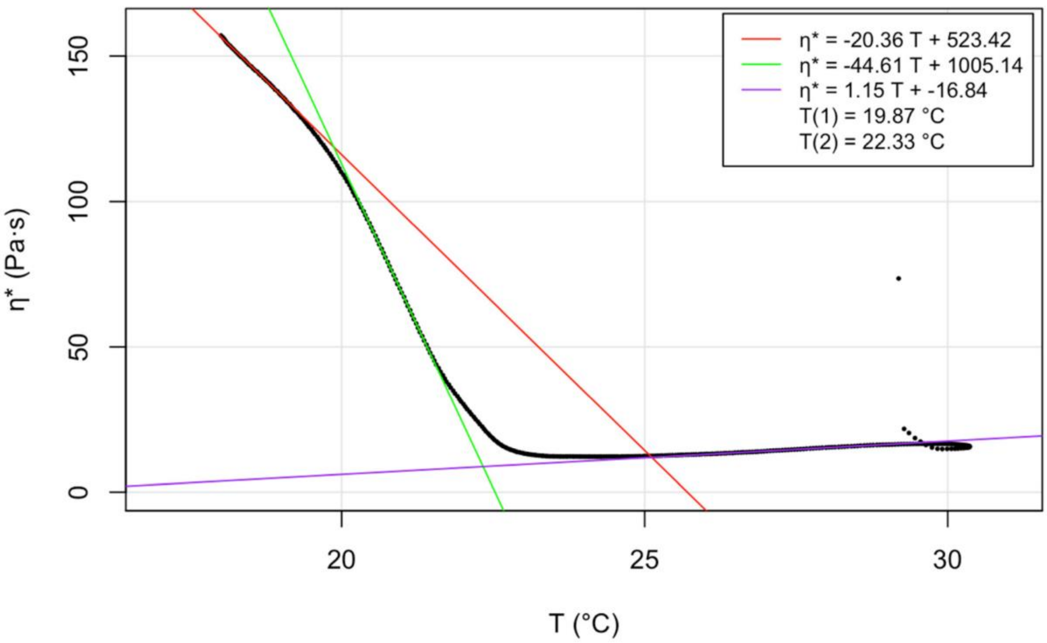

Figure S4. Temperature dependence of complex viscosity of DMPC (cooling speed 1 °C/min) -parallel measurement

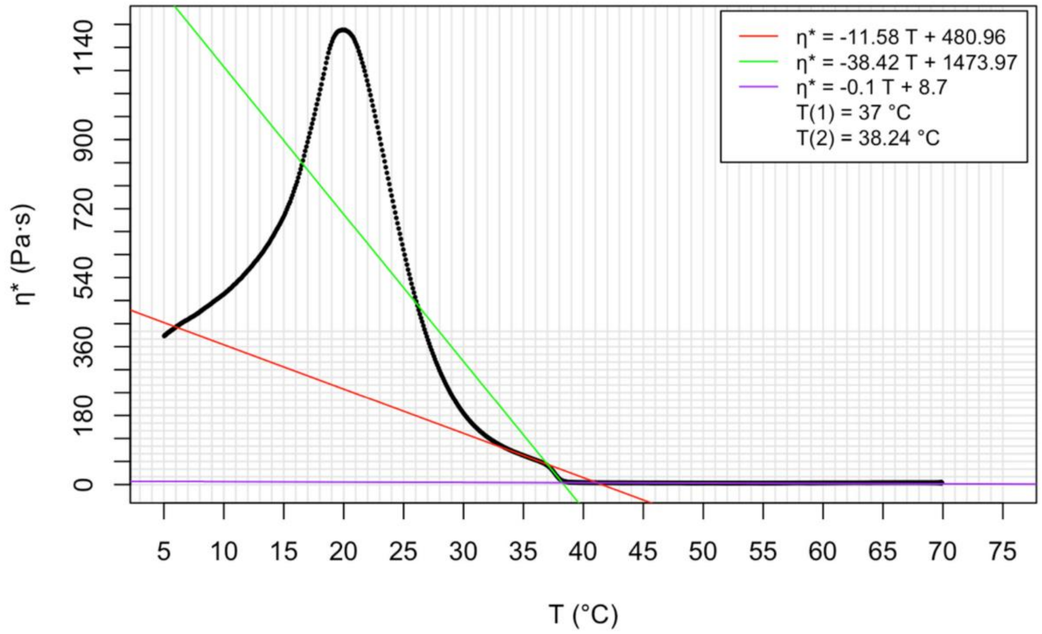

Figure S5. Temperature dependence of complex viscosity of DPPC (cooling speed 3 °C/min) - parallel measurement

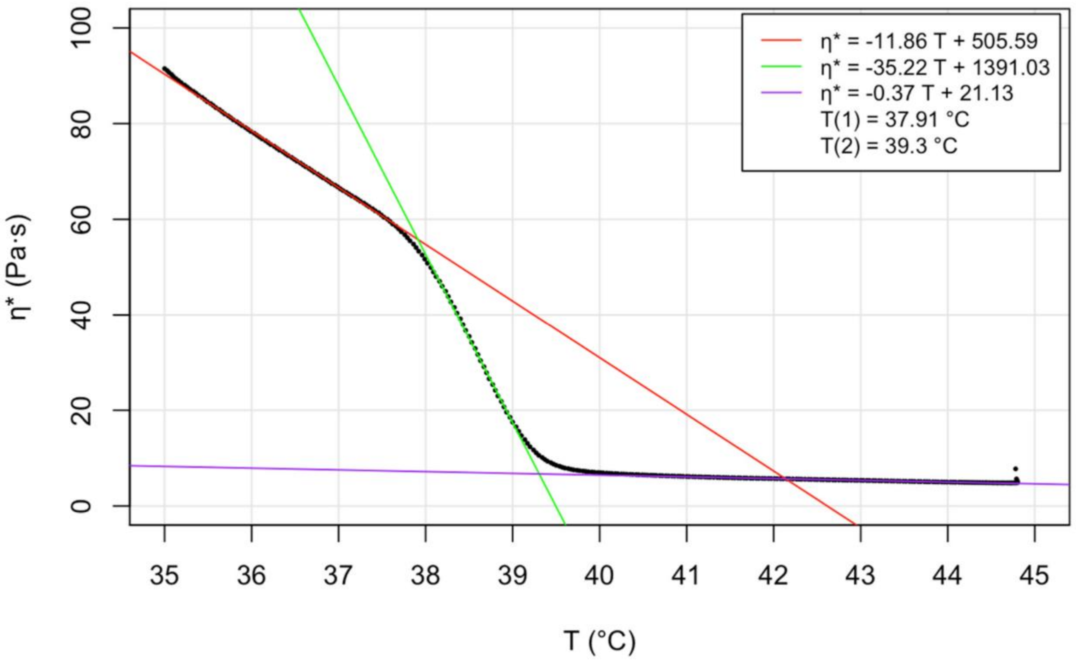

Figure S6. Temperature dependence of complex viscosity of DPPC (cooling speed 1 °C/min) -parallel measurement

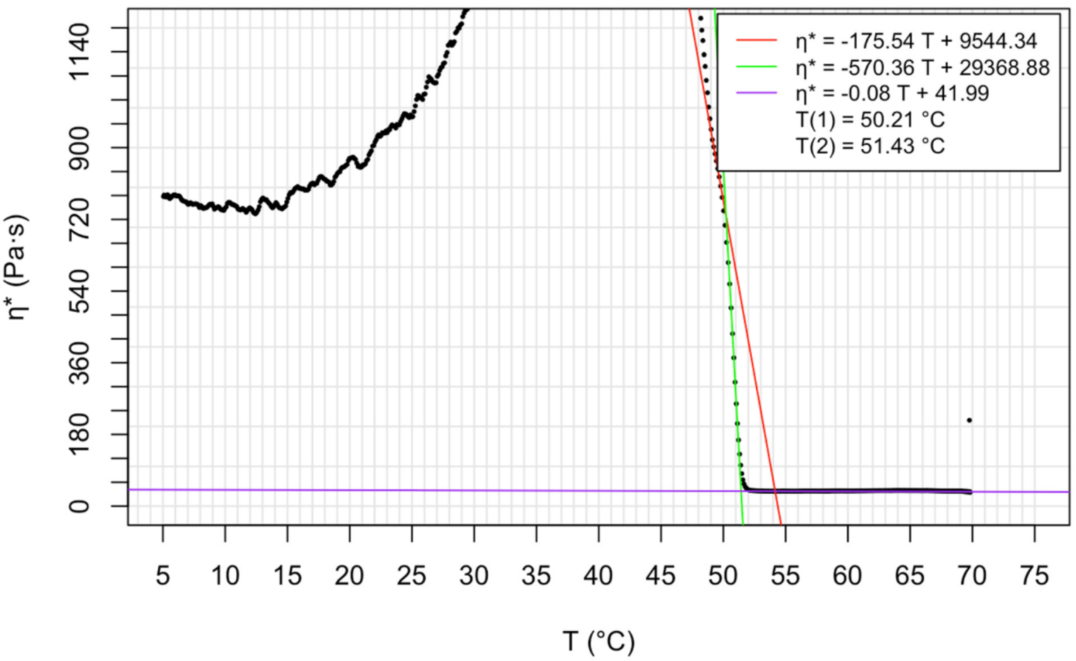

Figure S7. Temperature dependence of complex viscosity of DSPC (cooling speed 3 °C/min) - parallel measurement

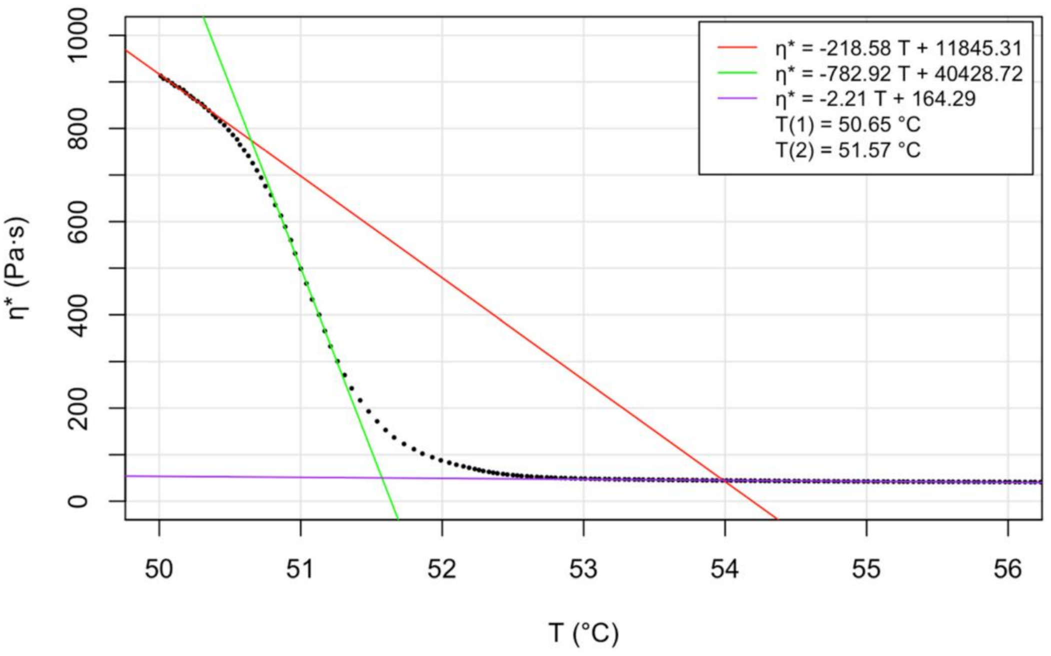

Figure S8. Temperature dependence of complex viscosity of DSPC (cooling speed 1 °C/min) -parallel measurement

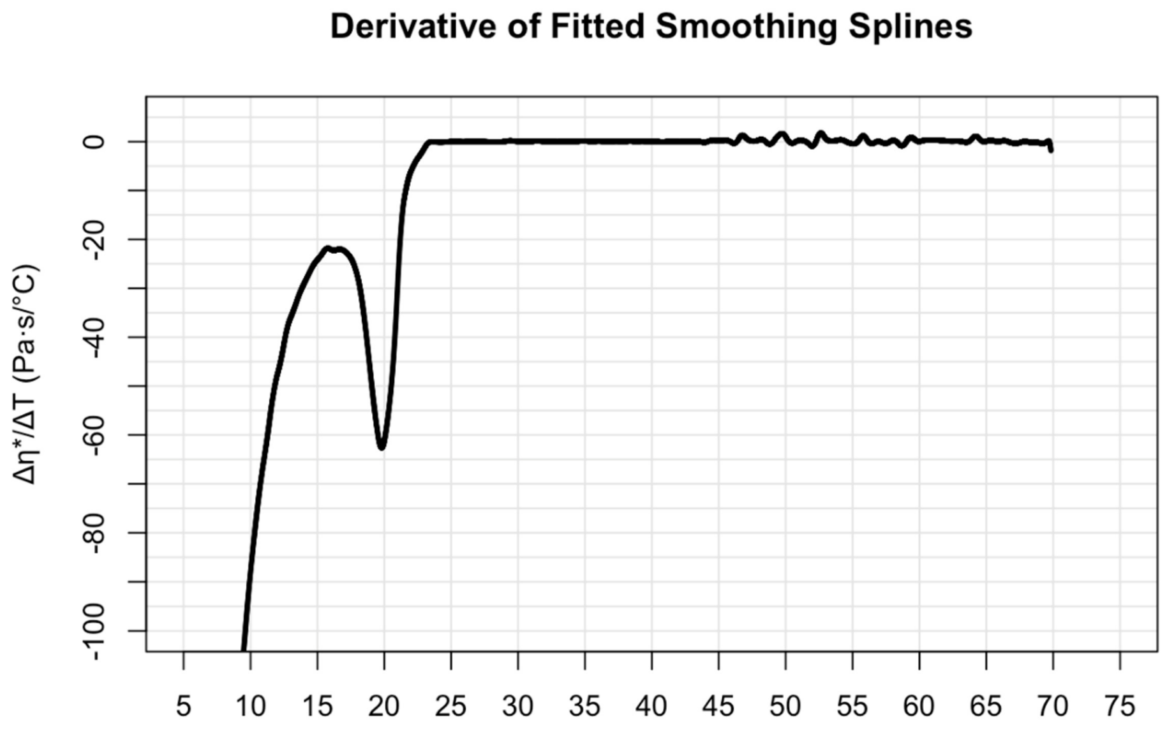

Figure S9. First derivative of the temperature-dependent complex viscosity of DMPC at 3 °C/min cooling rate

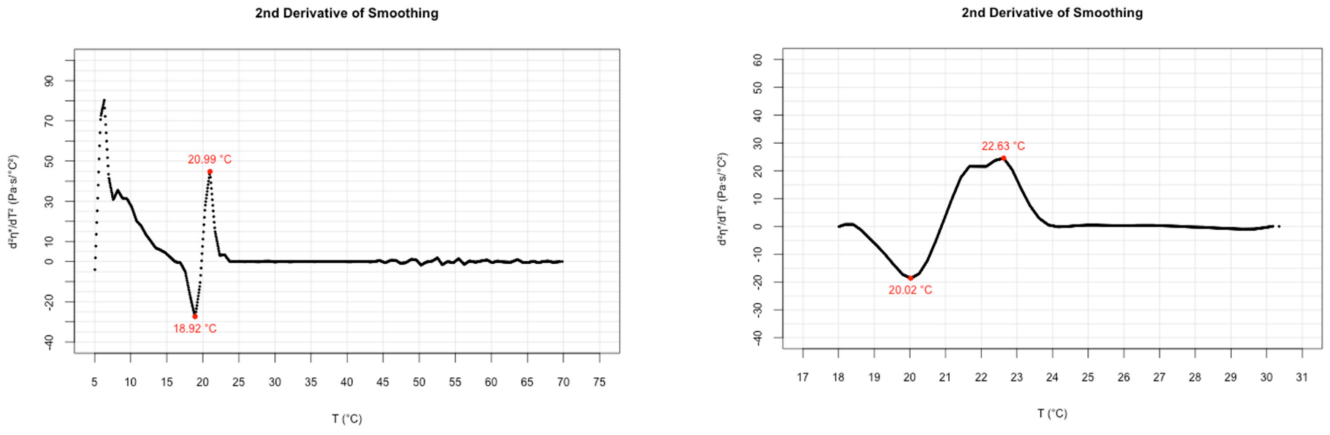

(a) (b)

Figure S10. Second derivative of the temperature-dependent complex viscosity of DMPC parallel measurement (a) at 3 °C/min cooling rate; (b) at 1 °C/min cooling rate;

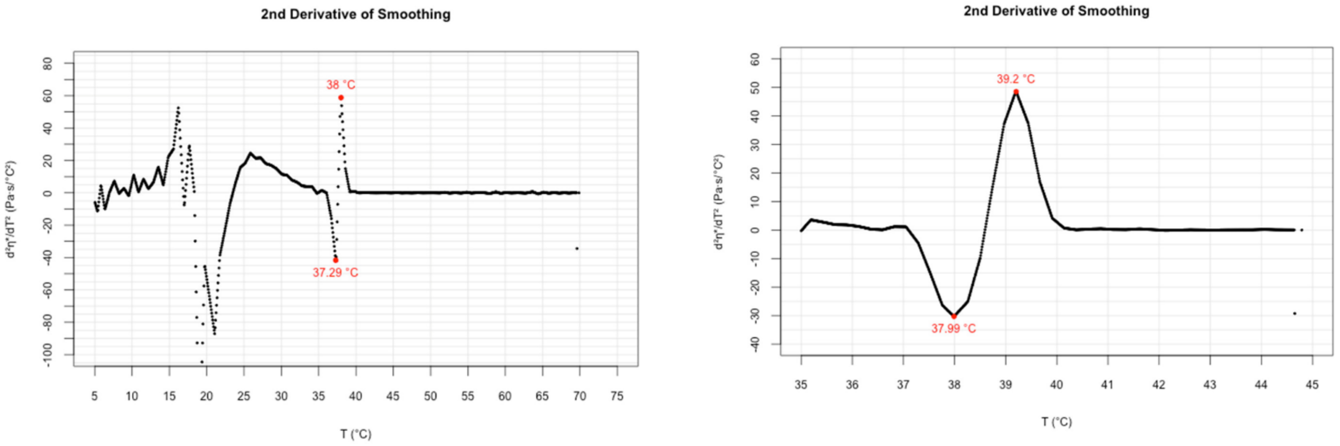

(a) (b)

Figure S11. Second derivative of the temperature-dependent complex viscosity of DPPC parallel measurement (a) at 3 °C/min cooling rate; (b) at 1 °C/min cooling rate;

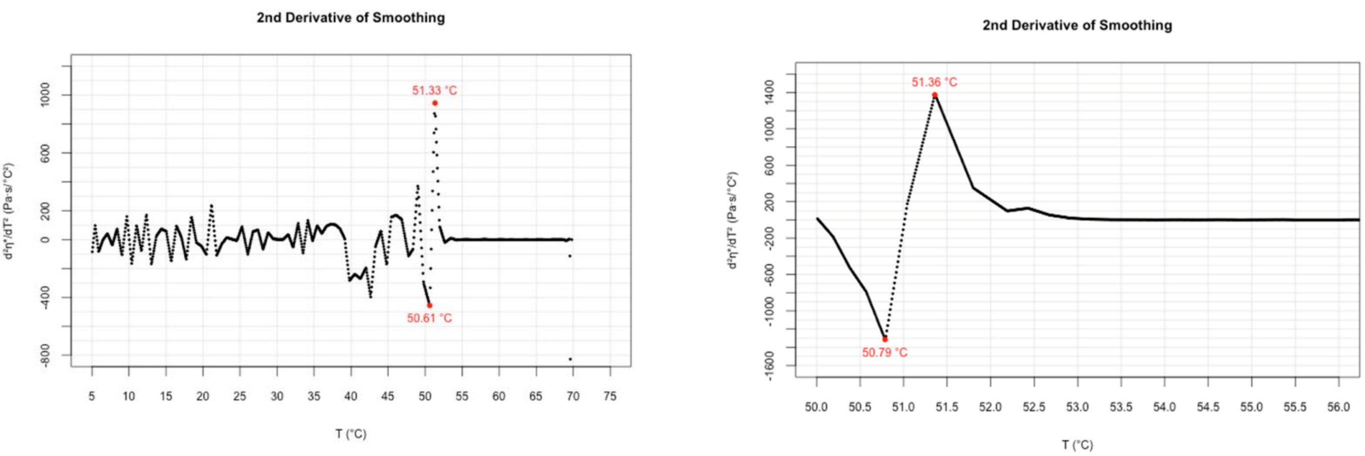

(a) (b)

Figure S12. Second derivative of the temperature-dependent complex viscosity of DSPC parallel measurement (a) at 3 °C/min cooling rate; (b) at 1 °C/min cooling rate;
